# Development of an electronic health record-based Human Immunodeficiency Virus (HIV) risk prediction model for women, incorporating social determinants of health

**DOI:** 10.1186/s12889-025-23460-2

**Published:** 2025-07-02

**Authors:** Yiyang Liu, Aokun Chen, Hwayoung Cho, Khairul A. Siddiqi, Robert L. Cook, Mattia Prosperi

**Affiliations:** 1 Department of Epidemiology, College of Medicine and College of Public Health and Health Professions, University of Florida, 2004 Mowry Road, PO Box 100231, Gainesville, FL 32610-0231 USA; 2 Department of Health Outcomes & Biomedical Informatics, College of Medicine, University of Florida, Gainesville, FL USA; 3 Department of Family, Community and Health System Science, College of Nursing, University of Florida, Gainesville, FL USA; 4 Department of Community and Behavioral Health, Elson S. Floyd College of Medicine, Washington State University, Spokane, Washington, USA

**Keywords:** HIV, Risk prediction model, Women, Social determinants of health, Machine learning

## Abstract

**Background:**

Human Immunodeficiency Virus (HIV) pre-exposure prophylaxis (PrEP) prevents HIV transmission but has low uptake among women. Identifying women who could benefit from PrEP remains a challenge. This study developed a women-specific model to predict HIV risk within a year using electronic health record (EHR) data and social determinants of health (SDoH).

**Methods:**

We conducted a case-cohort study using EHR and claims data from a centralized patient repository in the Southeastern United States (OneFlorida+). The dataset was split into 60% training, 30% testing, and 10% calibration. Five-fold cross-validation was applied for hyperparameter tuning. Contextual-level SDoH were linked to EHR/claim data. Various machine learning (ML) methods were tested, and Shapley Additive Explanations (SHAP) values were used to interpret the model.

**Results:**

Our sample included 1,458 women newly diagnosed with HIV and 33,155 controls who had never been diagnosed. The XGBoost model outperformed other ML methods, achieving an area under the curve (AUC) of 89.3%. Sensitivity and specificity ranged from 83% to 82% at the optimal Youden’s index cutoff, identifying 20% as high risk, to 42% and 97% at the optimal F1 score cutoff, identifying 5% as high risk. Of the 20 features with the highest SHAP values, 11 were related to SDoH.

**Conclusion:**

The final model, incorporating demographics, clinical features, and SDoH, can predict HIV risk in the next year for women. Several SDoH factors were found to be important predictors. Future work could involve stakeholders in implementing the model into HIV PrEP decision support and exploring causal pathways to guide risk-reduction interventions.

**Supplementary Information:**

The online version contains supplementary material available at 10.1186/s12889-025-23460-2.

## Introduction

Over 7,000 women were newly diagnosed with Human Immunodeficiency Virus (HIV) in 2022, which corresponds to 18% all new diagnoses in the United States (US) [1]. HIV pre-exposure prophylaxis (PrEP) can prevent HIV transmission up to 100% in women [2]. Currently, there are two Food and Drug Administration (FDA) approved PrEP options for women: once-daily oral pills (TDF/FTC) and long-acting injectables (CAB-LA). A new LA PrEP medication, lenacapavir (LEN), requiring an injection every six months is in the pipeline and has shown impressive efficacy among women in one study, with zero HIV infections reported during follow-up [3]. Unfortunately, PrEP uptake remains disproportionately low among women relative to men [4, 5]. Despite accounting for 20% of new HIV diagnoses in the United States, women make up only 7% of PrEP users [6, 7].

One significant barrier to promoting PrEP uptake by women is the difficulty in identifying those who may benefit from it [8]. The Centers for Disease Control and Prevention (CDC) guidelines [9], which require women to recognize their partner’s HIV risk or detect asymptomatic sexually transmitted infections (STIs), may not be sufficient for identifying all women at risk for HIV infections (Calabrese et al., 2019; Turner, Roepke, Wardell, & Teitelman, 2018). As a result, many women with existing HIV risk exposure and willingness to use PrEP are not being recognized as eligible [10]. Providers also face challenges in assessing women’s HIV risks due to time constraints [11] and biases, such as underestimating women’s vulnerability to HIV [12–14]. These factors may contribute to inadequate HIV preventive utilization among women. An HIV risk prediction model, incorporated into electronic health record (EHR) systems, can help providers better identify women at risk of HIV and promote the use of HIV preventive services [15].

Recent literature has demonstrated the potential of EHR-based automated HIV risk prediction models in helping providers better identify individuals who may benefit from HIV prevention services [16–18]. However, past prediction models that were mostly effective for men have been less successful in predicting HIV risk among women, in part due to male-focused predictors (e.g., men who has sex with men) and limited representation of women in study samples [16–18]. Contextual and individual level social determinants of health (SDoH) that could be key HIV risk factors for women [19–21] were not accounted for in those models. In recent years, women-specific prediction models have been developed. Some models included several women-specific HIV predictors, such as pregnancy [22], clinical diagnosis and laboratory evidence of trichomonas [23], or used data-driven approaches to identify important clinical predictors [24]. The application of these models still faces limitations such as moderate model performance [22], poor generalizability due to small sample sizes of women with outcomes [22, 23], and challenges in interpreting the identified predictors [24]. Additionally, these HIV risk prediction models, including those focused on men, considered no or only a limited number of SDoH (e.g., neighborhood deprivation, received care in high-HIV-burden areas) [18, 23].

In this study, we developed a women-specific machine learning (ML) HIV risk prediction model using EHR and claims data from a large clinical research network in the southern US, a region disproportionately affected by HIV [1]. We incorporated a wide range of contextual-level SDoH, linked to patient-level EHRs via residential history and ZIP code. We expect that the inclusion of SDoH will improve the performance of HIV risk prediction model.

## Methods

### Data sources and study design

The OneFlorida+ (OneFL) Data Trust is a centralized research patient data repository, created and managed by the OneFL Clinical Research Consortium [25]. It contains longitudinal EHRs and claims data since 2012 harmonized using the Patient-Centered Outcomes Research Institute (PCORI) common data model (PCORnet CDM). The repository includes data from 13 health systems in Florida, fully covering the 7 Ending the HIV Epidemic (EHE) Priority Counties in the state, as well as 1 health system in Georgia and 1 in Alabama. We requested a limited dataset from OneFL where all 16 protected health information (PHI) attributes defined by the Health Insurance Portability and Accountability Act (HIPAA) Privacy Rule are removed, except for dates and zip codes. In addition, OneFL has established an exposome data linking individual-level EHR with various community-level factors (including environmental exposure and SDoH) using patients’ residential history and ZIP code [26].

We designed a case cohort study to predict a new HIV diagnosis (outcome) within a year of assessing HIV risks. New HIV diagnosis was identified based on the first evidence of HIV infection, defined as the presence of an HIV diagnosis code, laboratory results suggestive of HIV, or prescriptions for HIV treatment, among patients with at least one year of EHR data, one year of continuous insurance enrollment, and a minimum of three clinical encounters prior to the first HIV-related record. The flow chart of sample selection is shown in Appendix Fig. 1. Eligibility criteria included female sex, age 18 or older and at least one year of prior EHR data available during the sample selection year. Cases included women newly diagnosed with HIV (incidence) [27, 28]. Controls were randomly selected from patients who met the eligibility criteria but had not been diagnosed with HIV. We “recruited” (observationally) patients annually from 2013 to 2022. Cases entered the study in the year of their HIV diagnosis, and controls entered in any year they had at least one encounter. Controls were selected only once, even if they met the criteria in multiple years. The index date for both cases and controls were set as January 1 of the calendar year they entered the study.

### Ethics approval

This secondary data analysis of a limited dataset was exempt approved by the University of Florida Institutional Review Board (IRB202302118), with the need for informed consent waived.

### Candidate predictors

We considered a wide range of candidate predictors from four domains: (1) demographics; (2) expert-selected variables; (3) clinical variables selected by a frequency filter; and (4) contextual-level SDoH. Demographics include age, race, and ethnicity; age is encoded as continous and race/ethnicity is encoded as binary variables (i.e., one-hot). For expert-selected variables, we included predictors identified from previously published HIV risk prediction models (such as insurance/payer, STIs, viral hepatitis, mental health, and substance) and additional clinical factors not considered by past models (e.g., human papillomavirus (HPV) infection, pelvic inflammatory disease, urinary tract infection, and contraceptive use). The full list of variables included and their corresponding ICD-9 and 10 codes used are detailed in Appendix Table 1.

We mapped all ICD-9 and ICD-10 codes to Phecodes, a hierarchical and standardized classification system that clusters disease diagnosis and service codes into broader phenotypic categories [29]. A frequency filter was then applied to identify all Phecodes with a prevalence greater than 3% in the study sample (such as chronic pain, hyperlipidemia). For each expert-selected and frequency-filtered clinical variable, two binary variables were created to represent the presence or absence of the condition: one for the past 12 months before the index year, and the other for lifetime but not in the past 12 months.

From OneFL exposome data, we requested all variables associated with SDoH, such as neighborhood deprivation, various crime rates, walkability, food access, and social capital. Additionally using the received ZIP code, the research team linked received OneFL data with Florida Drug-Related Outcomes Surveillance and Tracking (FROST) [30], AIDSVu [31], and FLHealthCHARTS [32] to obtain county-level data on substance use related death rate, HIV and STI incidence and prevalence, and other SDoH (e.g., income, housing, and insurance). The full list of exposome variables included is listed in Appendix Table 2. An average value was calculated if multiple years of data was available. SDoH exposome variables were continuous and were standardized using z-score scaling to have mean of 0 and standard deviation of 1.

Mean imputation was used for missing exposome due to missing ZIP code information to retain sample size and maintain consistency across models. As these variables represent aggregated community-level metrics rather than individual characteristics, mean imputation is a reasonable approach that minimizes bias while preserving interpretability and model stability. Most clinical variables were binary coded (1 = presence of diagnosis codes, 0 = absence), and no missing data handling was required.

To avoid multicollinearity, we removed one variable from each pair of highly correlated predictors (correlation > 0.80). Most variables removed are exposome variables. Nine frequency-filtered clinical variables were removed due to high correlations with expert-selected factors.

### Model development and evaluation

The model development and assessments were performed in accordance with the Transparent Reporting of a multivariable prediction model for Individual Prognosis Or Diagnosis (TRIPOD) guideline [33]; the checklist is provided as supplementary material.

A logistic regression model with demographic and expert-selected factors was used as the baseline model. This model was compared with ML methods using combinations of variable set: (1) only demographic features, (2) only expert-selected features, (3) only frequency-filtered clinical features, (4) only exposome features, (5) demographics plus expert-selected features, and (6) all features. We included 6 ML methods in this comparison, which include: Least Absolute Shrinkage and Selection Operator (LASSO) logistic regression, eXtreme Gradient Boosting (XGB), Support Vector Classification (SVC), Random Forest (RF), and Light Gradient Boosting Machine (LGBM).

We adopted a three-way dataset split, reserving 60% for model training, 30% for testing, and 10% for calibration. Hyperparameter tuning was performed using a randomized search and 5-fold cross-validation within the training split. To address the potential issue of imbalanced outcome (i.e., rare incidence of HIV diagnoses in the cohort), we considered class weights as one of the hyperparameters. Model performance was evaluated on the testing dataset using: Area Under the Curve (AUC) of the Receiver Operating Characteristic (ROC), sensitivity (i.e., recall), specificity, precision (i.e., positive predicted value, PPV), negative predicted value (NPV), and F1 score (2·precision·recall/(precision + recall)). Performance was also evaluated across different racial, ethnic, and age groups. AUCs were statistically compared using the Delong test [34, 35].

Finally, we calibrated the best-performing model using Platt scaling and evaluation of the Brier score—a metric ranging from 0 to 1 that measures the accuracy of probabilistic predictions, where 0 represents perfect predictions. This process adjusts the model’s predicted probabilities to better reflect the true likelihood of the outcome. After calibration, we evaluated various classification thresholds, including those that maximize Youden’s Index, the F1 score, and cutoffs for the top 5%, 10%, 15%, and 20% of the risk. For each threshold, we calculated absolute risk (predicted probability), sensitivity, specificity, PPV, and NPV.

Feature importance was evaluated using the mean and median SHAP values [36] and visualized with the SHAP Beeswarm plots. Features were ranked using two criteria: (1) by mean SHAP values, which highlight the features with the highest overall contribution to the model across all predictions, and (2) by maximum SHAP values, which emphasize features that, while potentially rare, had a significant impact on individual predictions.

## Results

### Study sample characteristics

A total of 1,458 women who were newly diagnosed with HIV (cases) and 33,155 women without HIV diagnosis (controls) were included in the study sample; therefore the proportion of HIV incident cases within a year from the encounter was 4.2%, and the control: case ratio was about 23:1. The demographic and select clinical features are listed in Table 1. The mean age in the cases was 43.7, younger than the mean age of 55.4 in the control groups. 20% of the cases and 51.9% of the controls were non-Hispanic White, 62.6% of cases and 23.8% of controls were non-Hispanic Black, 15.0% of cases and 18.9% of controls were Hispanic. Relative to controls, lower proportions in cases had Medicare (7.0% in cases vs. 14.6% in controls) or private (9.8% vs. 24.4%) as the primary insurance payer and a higher proportion had Medicaid (10.8% vs. 4.8%). Many of the clinical features selected by experts showed low prevalence.

**Table 1:** Study sample characteristics

Socio-Demographic	Cases, *n* = 1,458 Mean (SD)/ *n* (%)	Controls, *n* = 33,155 Mean (SD)/ *n* (%)
Mean age (SD)	43.7 (14.2)	55.4 (16.1)
Age group (%)		
13–29	720 (20.2)	3,244 (5.9)
30–44	1,015 (28.4)	9,919 (18.1)
45+	1,834 (51.4)	41,555 (75.9)
Race and ethnicity (%)		
Non-Hispanic White	305 (20.9)	17,198 (51.9)
Non-Hispanic Black	912 (62.6)	7,875 (23.8)
Hispanic	218 (15.0)	6,265 (18.9)
Other	23 (1.6)	1,817 (5.5)
Primary insurance payer (%)		
Medicare	103 (7.0)	4,834 (14.6)
Medicaid	158 (10.8)	1,586 (4.8)
Other governmental	62 (3.6)	722 (2.2)
Private	143 (9.8)	8,073 (24.4)
Self-pay/no insurance	65 (4.5)	799 (2.4)
Unknown/Other	937 (64.3)	17,141 (51.7)
Number of emergency department visits in the past year (%)		
0 times	966 (66.3)	23,786 (71.7)
1–2 times	304 (20.9)	7,316 (22.1)
3 + times	188 (23.9)	2,053 (6.2)
Diagnosis in the past year (%)		
Chlamydia	4 (0.3)	80 (0.2)
Gonorrhea	3 (0.2)	21 (0.06)
Syphilis	3 (0.2)	17 (0.05)
HPV	10 (0.7)	183 (0.6)
Herpes	11 (0.8)	149 (0.5)
Trichomoniasis	17 (1.2)	82 (0.3)
Hepatitis B	6 (0.4)	63 (0.2)
Hepatitis C	25 (1.7)	332 (1.0)
Depression	114 (7.8)	3,295 (9.9)
Anxiety	84 (5.8)	3,868 (11.7)
Tobacco use disorder	155 (10.6)	2,063 (6.2)
Alcohol use disorder	62 (4.3)	465 (1.4)
Cannabis use disorder	42 (2.9)	231 (0.7)
Cocaine use disorder	66 (4.5)	169 (0.5)
Opioid use disorder	37 (2.5)	308 (0.9)

### Model comparison

**Fig. 1:**
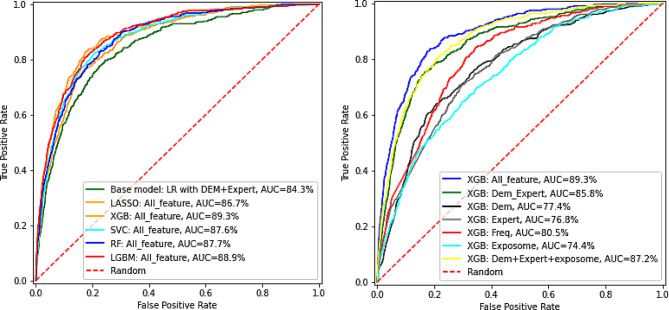
Receiver operating characteristic plot comparing the different machine learning methods (top) and variable subsets (bottom)

The base model, which used logistic regression and only included demographics and expert-selected clinical factors, achieved an AUC of 84.3%. Among all methods tested with the full set of predictors, LASSO achieved an AUC of 86.7%, while XGBoost performed best with an AUC of 89.3%, significantly outperforming both the base and LASSO models (both *p* < 0.001). Figure 1 shows the ROC curves for each model.

Using XGBoost, we evaluated predictive performance on models that used the variable (sub)sets on single/combined domains (Fig. 1). Besides the model fit on all variables, the best model was the one using demographics + exposome + expert-selected features with an AUC of 87.2%, followed by the one using demographics + expert-selected features with an AUC of 85.8%. Compared to the model using all variables, the other two models did not show a statistically significant difference in AUC (*p* = 0.982 and 0.053, respectively). Afterward, performance dropped significantly, with the frequency-filtered model (AUC 80.5%, *p* < 0.0001) and the demographics-only model (AUC 77.4%, *p* < 0.0001) showing statistically significant differences from the best model. The worst performance came from the exposome-only model, with an AUC of 74.4%.

**Fig. 2:**
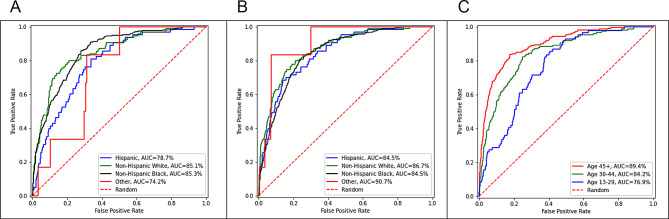
Receiver operating characteristic plot of XGBoost stratified by race/ethnicity and age group. Panel **A**. Stratified by race and ethnicity, including race and ethnicity as predictors. Panel **B**. Stratified by race and ethnicity, excluding race and ethnicity as predictors. Panel **C**. Stratified by age group

We then examined model performance stratified by race, ethnicity, and age groups (Fig. 2). Slightly higher AUC was observed among non-Hispanic Black (85.4%) and non-Hispanic White (85.7%) than Hispanic (79.3%) when race and ethnicity were included as the predictors. After excluding race and ethnicity as predictors, a higher model performance was observed across different racial/ethnic groups, with the exception of a minimal drop for non-Hispanic Black (84.7%). When stratified by age, the AUC ranged from 76.6% among people aged 13–29 to 89.4% among people aged 45+.

### Model calibration and threshold selection

The XGBoost model with all features was used as the final model, which was further calibrated using Platt scaling. Calibration helps align predicted probabilities more closely with actual outcomes. After calibration, the Brier Score improved from 0.08 to 0.03, indicating a closer match between predicted and observed probabilities. Results from the calibration are shown in Appendix Fig. 3.

Using the calibrated model, we calculated the classification cutoff to (1) optimize Youden’s index, which considers both sensitivity and specificity, and (2) optimize the F1 score, which takes into account sensitivity and PPV (i.e., recall and precision). We also evaluated cutoffs that classify 5%, 10%, 15%, and 20% of people as high risk. The cutoff for the optimal Youden’s index classified individuals with a predicted probability > 3%, roughly corresponding to the cutoff that classified 20% of people. The cutoff for the optimal F1 score classified individuals with a predicted probability > 25.8%, corresponding to the cutoff that classified 5% of people. Sensitivity, specificity, PPV, and NPV for each cutoff point are listed in Table 2. All cutoff points achieved a very high negative predictive value (NPV > 0.97). Sensitivity was highest at 0.83 when 20% of the population was classified, and lowest at 0.42 when only 5% of the population was classified. Specificity and PPV were highest when 5% of the population was classified, with values of 0.97 and 0.36, respectively. These metrics decreased when 20% of the population was classified, with specificity dropping to 0.82 and PPV to 0.17.

**Table 2:** Sensitivity, specificity, positive predictive value (PPV) and negative predictive value (NPV) at multiple classification cutoff points

Cutoff point Prob. >	Proportion of pts flagged	Sensitivity (recall)	Specificity	PPV (precision)	NPV
3.0% (Optimal Youden’s index)	~ 20%	0.83	0.82	0.17	0.99
5.7%	~ 15%	0.74	0.88	0.21	0.99
11.2%	~ 10%	0.62	0.92	0.26	0.98
25.8% (Optimal F1 score)	~ 5%	0.42	0.97	0.36	0.97

### Feature importance analysis

**Fig. 3:**
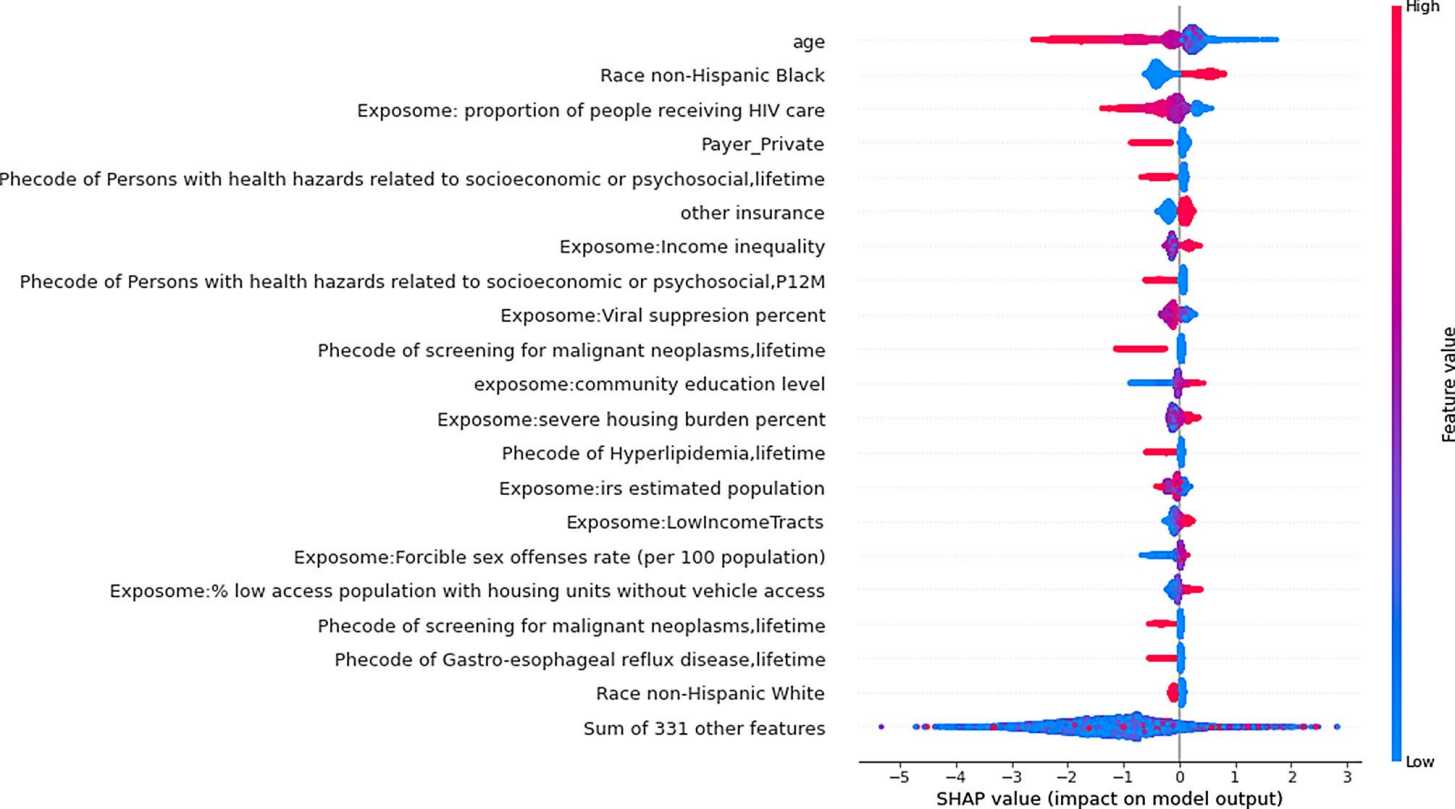
Top 20 Features with the highest mean SHAP values

Using mean SHAP values (Fig. 3), the top 20 most important features included demographic factors (age, race, and ethnicity), several contextual SDoH (e.g., neighborhood income inequality, education level, housing burden, low access to a vehicle), and factors directly related to community HIV risk (e.g., the proportion of people with HIV receiving HIV care, achieved viral suppression, and community sex offense crime rate). Individual level SDoH, identified with ICD codes in EHR, was also identified as an important predictor. Most of the features identified by maximum SHAP overlapped with those from the mean SHAP analysis (Appendix Fig. 4). Features including cocaine use disorder and lab tests for hepatitis were identified as important contributors to individual predictions based on maximum SHAP values but did not appear in the mean SHAP rankings. None of the clinical factors specifically selected for women, such as HPV infection or vaginal yeast infection, emerged as the most significant predictors.

The LASSO model was selected as an alternative due to its interpretability. Modeling results with LASSO are provided in the Appendix. Overall, the results were largely consistent with those of the XGBoost model, with significant overlap in the top 20 features identified by both models.

## Discussion

In this work, we used a large set of EHR and medical claims data with additional information on SDoH variables to develop an HIV risk prediction model for women. We included a wide range of women-specific HIV risk and contextual-level SDoH as candidate predictors. Our final model to differentiate women with new HIV diagnosis from controls achieved an AUC of 89.3%, which is higher than past EHR-based HIV prediction models, including models focused on women [16–18, 22–24].

Our findings underscore the importance of incorporating SDoH in HIV risk prediction, with 11 of the top 20 features being SDoH factors at both individual and contextual levels. SDoH information, especially at the contextual level, can be extracted from external data sources and linked to patient’s EHR to provide valuable insights into identifying individuals at risk. Incorporating SDoH into HIV risk prediction models allows the model to capture both individual-level clinical factors and the broader social and structural conditions that drive HIV risk. Although we expected that the inclusion of contextual-level SDoH would improve model performance, the observed improvement in discrimination (AUC 86.7% vs. 87.2%) was marginal and non-statistically significant (*p* = 0.982). This may because the baseline model is already performing well, and adding new features, even if informative, may not shift the curve significantly. Besides, people with disadvantaged contextual SDOH may also have other suboptimal clinical characteristics captured by clinical features in the model, meaning these SDOH features don’t provide entirely new discriminative power.

Interestingly, several well-known clinical risk factors for HIV, such as STI diagnoses and other risk factors specifically selected for women, did not rank among the top features. This could be due to the low prevalence of these factors in our dataset, which may have limited their influence on feature ranking despite their predictive strength. Additionally, important behavioral risk factors, such as number of sex partners, use of condoms, and risk characteristics of their sexual partners were not available in EHR data. Past work also demonstrated that relying solely on clinical factors listed in the CDC guidelines to identify candidates for HIV PrEP and routine testing may miss a large proportion of individuals who could potentially benefit from these prevention services [10, 28]. It is also important to note that the top predictors identified are based on their predictive power and do not imply causal relationships. Future research should consider using causal inference frameworks to explore underlying pathways and identify actionable interventions to reduce HIV risk.

The inclusion of contextual-level SDoH could also be valuable when implementing the prediction model at the point-of-care, particularly in supporting shared decision-making about HIV prevention services [16, 37]. Given that stigma was often associated with HIV [38], patients might feel judged if their HIV risk prediction were based solely on their personal medical or sexual history. By incorporating community-level risk factors and SDoH, providers can offer a more balanced and less judgmental explanation when communicating with the patients about their risks based on the prediction models. Future research could include healthcare providers’ perspectives on leveraging SDoH in risk models to aid them in initiating conversations about HIV prevention and whether this approach helps patients be more receptive to the results from HIV prediction models.

XGBoost has been widely used in developing clinical decision support systems [39, 40] and demonstrated the best performance among the ML models, which is consistent with prior studies [23, 24]. The use of SHAP values improves model interpretability and supports future real-world deployment by providing individualized explanations of risk predictions. The model performed better for older adults than for young adults. This may be due to the overrepresentation of older adult women in the study cohort, as our inclusion criteria required one year of prior EHR data and continuous insurance claims, which older individuals were more likely to meet. Race and ethnicity, as social constructs, were included in our final model. However, we observed that excluding them led to more equitable model performance across different racial and ethnic groups but slightly decreased AUC among non-Hispanic Black. These changes in AUC suggest that race and ethnicity may interact with other clinical and SDoH features, especially among non-Hispanic Black. Excluding race/ethnicity as predictor may risks masking disparities from potential structural inequities. This analysis highlighted the trade-off between fairness and the need to capture population-specific risk factors. Future studies are warranted to further examine model fairness and different social constructs.

We explored several classification thresholds. Higher thresholds (e.g., flagging 20% of patients) increase sensitivity but reduce PPV and specificity, potentially leading to more patients being flagged and increased alert fatigue when implemented as clinical decision support [41]. Lower thresholds (e.g., flagging 5% of patients), reduce false positives but increase the risk of missing individuals at risk (higher false negatives). Using our model, in a clinic with 1000 patients, with a 20% flagging threshold, the provider would need to counsel 200 patients to identify 34 true positives (200*0.17) but missing 7. In other words, providers would need to counsel about 6 patients (200/34) to identify one true positive. In contrast, if the threshold changed to 5%, flagging only 50 patients, 18 (50*0.36) true positives would be identified, but 24 would be missed. In this scenario, providers would need to counsel only 3 patients (50/18) to identify one true positive. Across all thresholds, PPVs are generally low due to the impact of disease prevalence on predictive value. In clinical settings, this means that while many individuals classified as high risk may not acquire HIV in the next year, the model can still be valuable for identifying subset of patients that may benefit a discussion about HIV prevention. Before incorporating the prediction model into clinical decision support, medical providers should be engaged to select appropriate thresholds based on the tradeoff between false positive and negative.

Our study has some limitations. First, our prediction model cannot identify individuals at risk of HIV if the diagnosis occurs during their first or second clinical encounter, as prior medical records are required for prediction. Additionally, we included limited individual-level SDoH variables. While disadvantaged individual-level SDoH were extracted from structured EHR using ICD Z codes and were shown to be important predictors, these codes are often underutilized and inadequately documented in EHRs [42]. A potential alternative is to use natural language processing (NLP) to extract more detailed SDoH information from clinical notes. Future research should explore whether incorporating additional individual-level SDoH can further enhance model performance. We reported calibrated predicted probabilities across various classification thresholds, but these estimates are subject to overestimation since the proportion of HIV cases in our study is higher than the true HIV incidence rate in the population. Finally, we did not perform external validation of our model, which is an important limitation. Future studies could assess model transportability using EHR data from other Florida health systems or from other states, with contextual-level SDoH linked through the same data pipeline.

## Conclusion

Our final XGBoost model, incorporating demographics, clinical features from EHR, and SDoH, can predict HIV risk in the next year for women. Several SDoH factors were found to be important predictors of HIV among women, such as community income, education level, and crime rate. Future work should engage healthcare providers, patients, and other stakeholders to identify appropriate ways to implement the prediction model into clinical decision support tools to help promote shared decision-making regarding the use of HIV PrEP and routine testing. Further research should also consider adopting causal frameworks to explore causal pathways from the prediction model and identify actionable interventions to reduce HIV risk.

## Electronic supplementary material

Below is the link to the electronic supplementary material.

Supplementary Material 1

## Data Availability

OneFlorida data used in the study can be requested at https://onefloridaconsortium.org/front-door/research-infrastructure-utilization-application/.

## Abbreviations

AUC: Area Under the Curve
CDC: Centers for Disease Control and Prevention
CDM: Common data model
EHE: Ending the HIV Epidemic
EHR: Electronic health record
FDA: Food and Drug Administration
FROST: Florida Drug-Related Outcomes Surveillance and Tracking
HIPAA: Health Insurance Portability and Accountability Act
HIV: Human immunodeficiency virus
HPV: Human papillomavirus
ICD: International Classification of Diseases
IRB: Institutional Review Board
LASSO: Least Absolute Shrinkage and Selection Operator
LGBM: Light Gradient Boosting Machine
ML: Machine learning
NPV: Negative predicted value
PCORI: Patient-Centered Outcomes Research Institute
PHI: Protected health information
PPV: Positive predicted value
PrEP: Pre-exposure prophylaxis
RF: Random Forest
ROC: Receiver Operating Characteristic
SD: Standard deviation
SDoH: Social determinants of health
SHAP: Shapley Additive Explanations
STI: Sexually transmitted infection
SVC: Support Vector Classification
TRIPOD: Reporting of a multivariable prediction model for Individual Prognosis Or Diagnosis
US: United States
XGB: eXtreme Gradient Boosting
