# Supplementary material for: Development of an electronic health record-based Human Immunodeficiency Virus (HIV) risk prediction model for women, incorporating social determinants of health
Source: BMC Public Health. 2025 Jul 2;25:2257. doi: 10.1186/s12889-025-23460-2 (PMC12219962; doi:10.1186/s12889-025-23460-2)
Supplement: Supplementary file 1 — Supplementary Material 1 [file 12889_2025_23460_MOESM1_ESM.docx]

Appendix documents

Appendix Table 1. ICD codes for extraction of expert-selected clinical factors

|  | ICD-9 | ICD-10 |
| --- | --- | --- |
| **Sexually transmitted infections (STIs)** |  |  |
| Chlamydia | 078.88, 079.98, 079.88, 099.5, 099.41 | A55, A56, A71, A74.0, A74.81, A74.89 |
| Gonorrhea | 097, 098, 099.0 | A54, O98.2 |
| Syphilis | 090, 091, 097.9 | A51, A52, A53.0, A53.9, O98.1 |
| Human papillomavirus (HPV) infection | 078.1, 078.5, 079.4, 796.75, 796.79, 795.05, 795.15, 795.19 | A63.0, B97.7, B97.81, R85.82, R85.618, R87.810, R87.811, R87.628 |
| Herpes | 053.9, 054.1, 054.9, 054.8, 054.79 | A60.0, A60.1, A60.9 |
| Unspecified viral infection | 079.99 | B97.89 |
| Trichomoniasis | 131 | A59 |
| Other STIs | 099.9 | A63, A64 |
| **Reproductive Health** |  |  |
| Pelvic inflammatory disease (PID) | 614 | N73, N70 |
| Bacterial Vaginosis (BV) | 616.1 | N76.0, N76.1 |
| Vaginal Yeast Infection | 112.1 | B37.3 |
| Pain in abdomen or pelvis | 789 | R10 |
| Urinary tract infection | 599 | N39.0 |
| Contraceptive Use | V25 | Z30 |
| **Viral** **hepatitis** |  |  |
| **Hepatitis B** | 070.2, 070.3, V02.61 | B16, B18.0, B18.1, B19.1, Z22.51 |
| **Hepatitis C** | 070.4, 070.5, 070.7, V02.62 | B17, B18.2, B19.2, Z22.52 |
| **Psychiatric conditions** |  |  |
| Depression | 296.2, 298.0, 311, 296.3 | F32, F33 |
| Anxiety | 300.0 | F14 |
| PTSD | 309.81 | F43.1 |
| ADHD | 314 | F90 |
| Bipolar | 296 | F31 |
| Stress reaction/disorder | 308, 309.82, 309.83, 309.89 | F43.0, F43.8, F43.9 |
| adjustment disorder | 309.0, 309.1, 309.2, 309.4 | F43.2 |
| **Substance use disorder** |  |  |
| Tabacco use disorder | 305.1 | F17 |
| Alcohol use disorder | 305.0, 303, 305.9 | F10, 291 |
| Cannabis use disorder | 305.2, 304.3 | F12 |
| Cocaine use disorder | 305.6, 304.2 | F14 |
| Opioid use disorder | 305.5, 304.0, 304.7 | F11 |
| Sedative use disorder | 305.4, 305.8, 304.1 | F13 |
| Amphetamine use disorder | 305.7, 304.4 | F15 |
| Hallucinogen use disorder | 305.3, 304.5 | F16 |
| Other substance use disorder | 305.9, 304.9, 304.8, 304.6 | F18, F19, 292 |
| **Chronic comorbidity** |  |  |
| Myocardial infarction | 410, 412 | I21, I22, I25.2 |
| Congestive heart failure | 428, 398.91, 402.01, 402.11, 402.91, 404.01, 404.03, 404.11, 404.13, 404.91,404.93, 425.4, 425.5, 425.6, 425.7, 425.8, 425.9 | I09.9, I11.0, I13.0, I13.2, I25.5, I42.0, I42.5, I42.6, I42.7, I42.8, I42.9, I43, I50, I29.0 |
| Peripheral vascular disease | 93.0, 437.3, 440, 441, 443, 447.1, 557.1, 557.9, V43.4 | I70, I71, I73.1, I73.8, I73.9, I77.1, I79.0, I79.2, K55.1, K55.8, K55.9, Z95.8, Z95.9 |
| Cerebrovascular disease | 362.34, 430, 431, 432, 433, 434, 435, 436, 437, 438 | G45, G46, H34.0, I60, I61, I62, I63, I64, I65, I66, I67, I68, I69 |
| Dementia | 290, 294.1, 331.2 | F00, F01, F02, F03, F05.1, G30, G31.1 |
| Chronic pulmonary disease | 416.8, 416.9, 490, 491, 492, 493, 494, 495, 496, 497, 498, 499, 500, 501, 502, 503, 504, 505, 506.4, 508.1, 508.8 | I27.8, I27.9, J40, J41, J42, J43, J44, J45, J46, J47, J60, J61, J62, J63, J64, J65, J66, J67, J68.4, J70.1, J70.3 |
| Rheumatic disease | 446.5, 710.0, 710.1, 710.2, 710.3, 710.4, 714.0, 714.1 , 714.2, 714.8, 725 | M05, M06, M31.5, M32, M33, M34, M35.1, M35.3, M36.0 |
| Peptic ulcer | 531, 532, 533, 534 | K25, K26, K26, K27, K28 |
| Mild liver disease | 70.22, 70.23, 70.32, 70.33, 70.44, 70.55, 70.6, 70.9, 570, 571, 573.3, 573.4, 573.8, 573.9, V42.7 | B18, K70.0, K70.1, K70.2, K70.3, K70.9, K71.3, K71.4, K71.5, K71.7, K73, K74, K76.0, K76.2, K76.3, K76.4, K76.8, K78.9, Z94.4 |
| Diabetes without chronic complication | 250.0, 250.1, 250.2, 250.3, 250.8, 250.9 | E10.0, E10.1, E10.6, E10.8, E10.9, E11.0, E11.1, E11.6, E11.8, E11.9, E12.0, E12.1, E12.6, E12.8, E12.9, E13.0, E13.1, E13.6, E13.8, E13.9, E14.0, E14.1, E14.6, E14.8, E14.9 |
| Diabetes with chronic complication | 250.4, 250.5, 250.6, 250.7 | E10.2, E10.3, E10.4, E10.5, E10.7, E11.2, E11.3, E11.4, E11.5, E11.7, E12.2, E12.3, E12.4, E12.5, E12.7, E13.2, E13.3, E13.4, E13.5, E13.7, E14.2, E14.3, E14.4, E14.5, E14.7 |
| Hemiplegia or paraplegia | 334.1, 342, 343, 344.0, 344.6, 344.9 | G04.1, G11.4, G80.1, G80.2, G81, G82, G83.0, G83.1, G83.2, G83.3, G83.4, G83.9 |
| Renal disease | 403.01, 403.11, 403.91, 404.02, 404.03, 404.12, 404.13, 404.13, 404.92,4 04.93, 582, 583.0, 583.1, 583.2, 583.3, 583.4, 583.5, 583.6, 583.7, 585, 586, 588, V42.0, V45.1, V56 | I12.0.4, I13.1, N03.2, N03.3, N03.4, N03.5, N03.6, N03.7, N05.2, N05.3, N05.4 , N05.5 , N05.6 , N05.7, N18, N19, N25, N49.0, N49.1, N49.2, Z94.0, Z99.2 |
| Malignant tumors | 140, 141, 142, 143, 144, 145, 146, 147, 148, 149,150, 151, 152, 153, 154, 155, 156, 157, 158, 159, 160, 161, 162, 163, 164, 165, 166, 167, 168, 169, 170, 171, 172, 174, 175, 176, 177, 178, 179, 180, 181, 182, 183, 184, 185, 186, 187, 188, 189, 190, 191, 192, 193, 194, 195, 200, 201, 202, 203, 204, 205, 206, 207, 208, | C00, C01, C02, C03, C04, C05, C06, C07, C08, C09, C10, C11, C12, C13, C14, C15, C16, C17, C18, C19, C20, C21, C22, C23, C24, C25, C26, C30, C31, C32, C33, C34, C37, C38, C39, C40, C41, C43, C45, C46, C47, C48, C49, C50, C51, C52, C53, C54, C55, C56, C57, C58, C60, C61, C62, C63, C64, C65, C66, C67, C68, C69, C70, C71, C72, C73, C74, C75, C76, C81, C82, C83, C84, C85, C88, C90, C91, C92, C93, C94, C95, C96, C97 |
| Moderate or severe liver disease | 456.0, 456.1, 456.2, 572.2, 572.3, 572.4, 572.5, 572.6, 572.7, 572.8 | I85.0, I85.9, I86.4, I98.2, K70.4, K71.1, K72.1, K72.9, K76.5, K76.6, K76.7 |
| Metastatic tumor | 196, 197, 198, 199 | C77, C78, C79, C80 |
| **Other diagnoses** |  |  |
| High-risk sexual behavior | V69.2 | Z72.5 |
| Adult sexual abuse | 995.83 | T74.21 |
| Domestic violence | 995.81 | T74.11 |
| Exposure to HIV or other viral disease | V01.79 | Z20.6 |
| Exposure to other STDs | V01.6 | Z20.2, Z20.6 |
| HIV screening | V73.89 | Z11.4 |
| STI screening | V74.5 | Z11.5 |
| other viral diseases screening | V73.99 | Z11.59 |
| HIV counseling | V65.44 | Z71.7 |

Lab testing (any testing and any testing with positive results): syphilis, gonorrhea, chlamydia, herpes *, trichomoniasis *, HPV *, substance use

Appendix Table 2. List of Exposome variables extracted

| Data source | Domain | Variable list | Geo-link level |
| --- | --- | --- | --- |
| American Community Survey (ACS) | Community Environment | neighborhood deprivation index, Unemployment rate, education attainment | Census Block Group level |
| Uniform Crime Reports (UCR) | Crime and Safety | Various crime rate per 100 population: aggravated assault, burglary, forcible sex offenses, larceny, murder, motor vehicle theft, robbery | Zip code level |
| National Walkability Index | Neighborhood Walkability | walkability | Census Block Group level |
| USDA Food Access Research Atlas (FARA) | Food Access | Percentage & number of low access population, of low income and low access population, of low access population with housing units without a vehicle, of children age 0-17 with low access, of seniors age 65+ with low access; Flag for low access tract using vehicle access and at 20 miles in rural areas, flag for tract where >=100 of households do not have a vehicle and beyond ½ mile from a supermarket, flag for low-income tract, flag for rural track | Census Tract level |
| United States Census Bureau | Social capital | Number of various establishments per 10,000 population: religious organizations, civic/social associations, business associations, political organizations, professional organizations, labor organizations, bowling centers, fitness/recreational sports centers, golf courses/country clubs, sports teams/clubs. | Zip code level |
| Florida Drug-Related Outcomes Surveillance and Tracking (FROST) | Substance use | County opioids death rate, benzodiazepine death rate, cocaine or psychostimulant death rate | County-level |
| AIDSVu | HIV, social determinants of health | Rates of Persons Using PrEP, Number of users, PrEP-to-Need-Ratio, Number of Persons Linked to HIV Care, Number of Persons who Received HIV Care, syphilis rate, poverty percent, high school education percent, median household income, income inequality, percent people without health insurance, percent unemployment, percent severe housing cost burden | County-level |
| FLHealthCHARTS | HIV | HIV prevalence rate, HIV incidence rate, rate of people with AIDS diagnosis | County-level |

Appendix Table 3. Sensitivity, specificity, positive predictive value (PPV) and negative predictive value (NPV) at multiple classification cutoff points.

| Cutoff point  Prob. > | Proportion of pts flagged | Sensitivity (recall) | Specificity | PPV (precision) | NPV |
| --- | --- | --- | --- | --- | --- |
| 6.0%  (Optimal Youden’s index) | ~20% | 0.74 | 0.84 | 0.17 | 0.99 |
| 7.3% | ~15% | 0.67 | 0.87 | 0.19 | 0.98 |
| 10.2% | ~10% | 0.53 | 0.92 | 0.23 | 0.98 |
| 15.1% (Optimal F1 score) | ~5% | 0.38 | 0.96 | 0.31 | 0.97 |

Appendix Table 4. Odds ratio and 95% confidence interval (CI) for the top 20 features from the LASSO model

| **Feature** | **Odds Ratio** | **Lower CI** | **Upper CI** |
| --- | --- | --- | --- |
| Age | 0.96 | 0.96 | 0.96 |
| Race non-Hispanic Black | 4.16 | 4.00 | 4.32 |
| Exposome: proportion of people receiving HIV care | 0.31 | 0.30 | 0.31 |
| Exposome: severe housing burden percent | 1.65 | 1.57 | 1.73 |
| Other insurance | 1.34 | 1.29 | 1.39 |
| Phecode of Persons with health hazards related to socioeconomic circumstances, lifetime | 0.47 | 0.46 | 0.49 |
| Phecode of Persons with health hazards related to socioeconomic circumstances, P12M | 0.49 | 0.47 | 0.51 |
| Phecode of screening for malignant neoplasms, lifetime | 0.35 | 0.33 | 0.37 |
| Long-term use of medications, lifetime | 0.43 | 0.41 | 0.45 |
| Exposome: Low Income Tracts | 1.25 | 1.21 | 1.29 |
| Private insurance | 0.41 | 0.40 | 0.43 |
| Exposome: Forcible sex offenses rate (per 100 people) | 1.28 | 1.23 | 1.32 |
| Phecode of Pain in joint, lifetime | 1.64 | 1.57 | 1.71 |
| Exposome: Viral suppression percent | 0.78 | 0.76 | 0.81 |
| Exposome: PrEP rates per 100k | 0.81 | 0.77 | 0.86 |
| Exposome: County Benzo death rate | 0.81 | 0.78 | 0.84 |
| Phecode of Hyperlipidemia, lifetime | 0.65 | 0.63 | 0.68 |
| Exposome: Unemployment percent | 0.82 | 0.79 | 0.85 |
| Exposome: Number of people with low food access | 0.78 | 0.73 | 0.83 |
| Exposome: People without insurance percent | 1.23 | 1.18 | 1.28 |

Appendix Figure 1. Flow chart of study sample identification


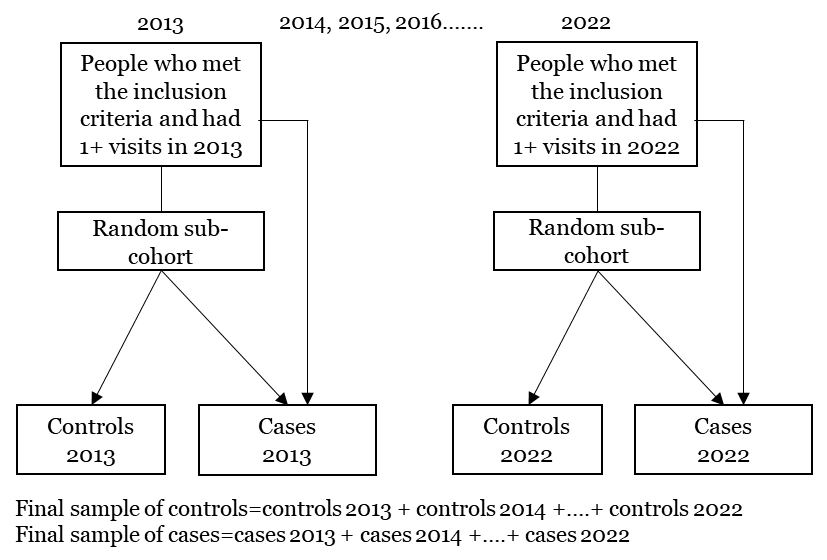


Appendix Figure 2. Extraction of predictors from EHRs


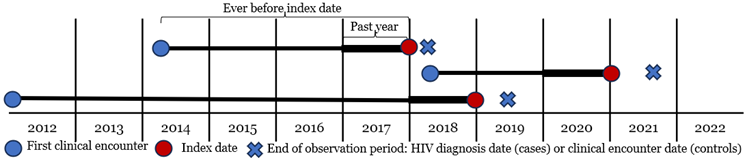


Appendix Figure 3. Calibration plot (left) and distribution of predicted probabilities (right) before and after calibration.

| 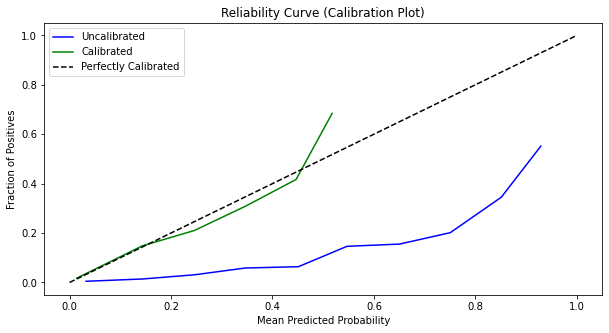 | 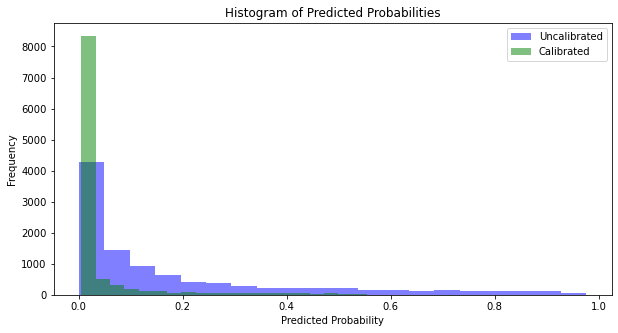 |
| --- | --- |

Appendix Figure 4. Top 20 Features with the highest maximum SHAP values for XGBoost model


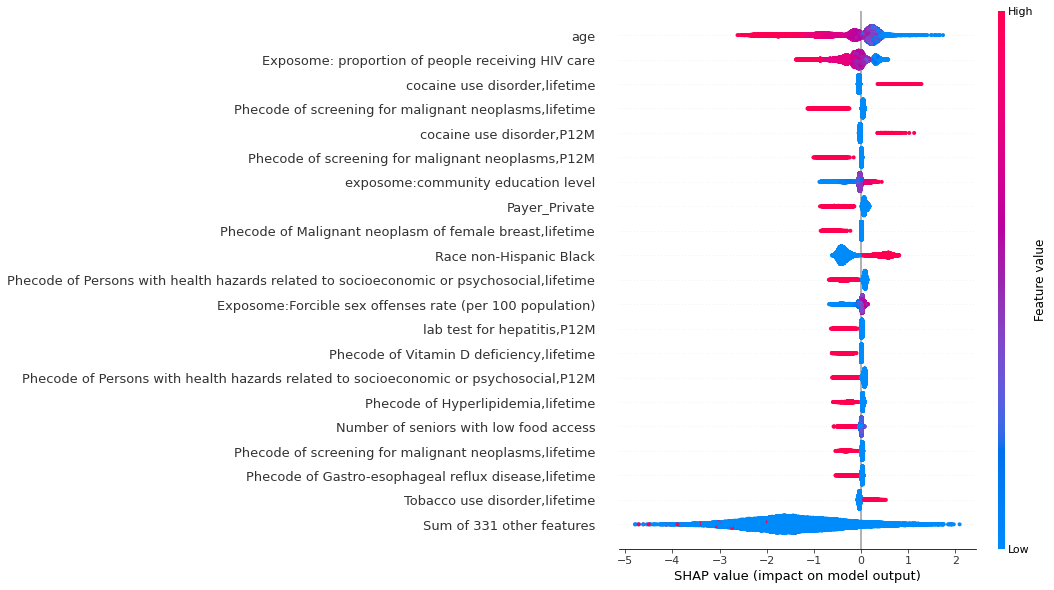


Appendix Figure 5. Receiver operating characteristic plot of LASSO using different variable (sub)sets.


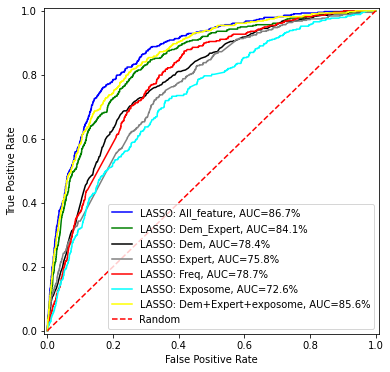


Appendix Figure 6. Receiver operating characteristic plot of LASSO stratified by race/ethnicity and age group.

| 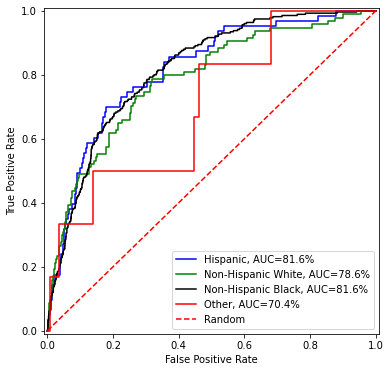 | 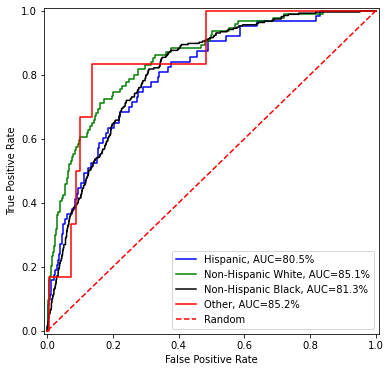 | 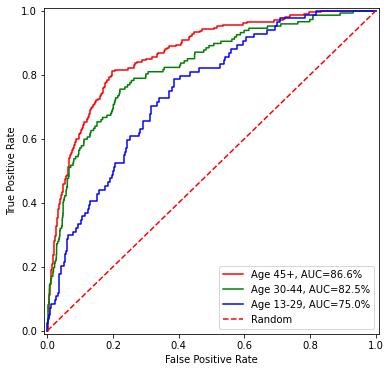 |
| --- | --- | --- |
| Panel A. Stratified by race and ethnicity, including race and ethnicity as predictors | Panel B. Stratified by race and ethnicity, excluding race and ethnicity as predictors | Panel C. Stratified by age group |

Appendix Figure 7. Top 20 Features with the highest mean SHAP values for LASSO


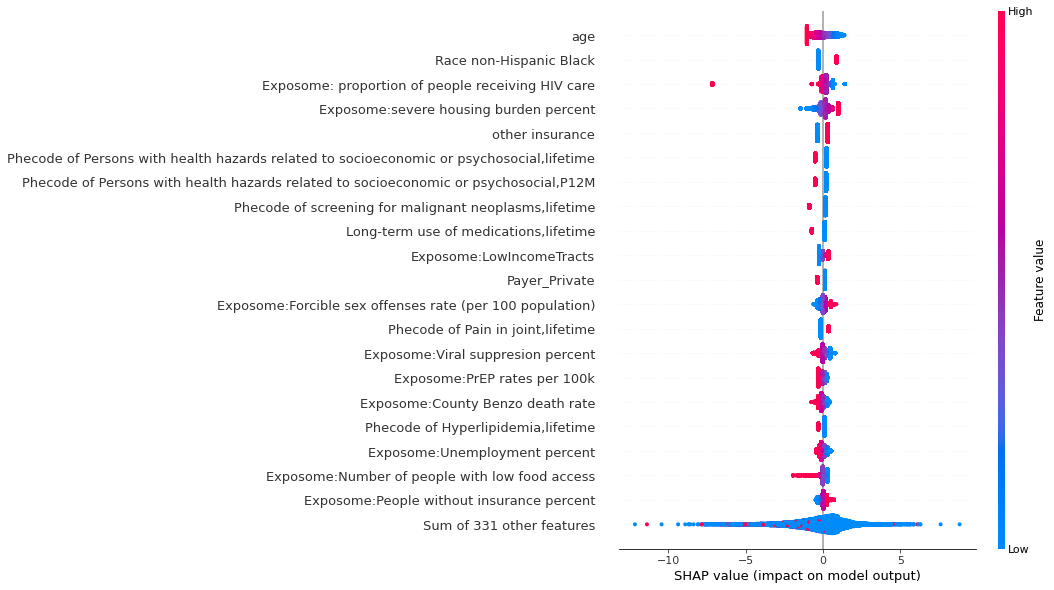


| TRIPOT checklist  **Section/topic** | **Item** | **Development or validation?** | **Checklist item** | **Page** |
| --- | --- | --- | --- | --- |
| **Title and abstract** |  |  |  |  |
| Title | 1 | D;V | Identify the study as developing and/or validating a multivariable prediction model, the target population, and the outcome to be predicted. | 1 |
| Abstract | 2 | D;V | Provide a summary of objectives, study design, setting, participants, sample size, predictors, outcome, statistical analysis, results, and conclusions. | 2-3 |
| **Introduction** |  |  |  |  |
| Background and objectives | 3a | D;V | Explain the medical context (including whether diagnostic or prognostic) and rationale for developing or validating the multivariable prediction model, including references to existing models. | 4 |
|  | 3b | D;V | Specify the objectives, including whether the study describes the development or validation of the model, or both. | 5 |
| **Methods** |  |  |  |  |
| Source of data | 4a | D;V | Describe the study design or source of data (e.g., randomized trial, cohort, or registry data), separately for the development and validation data sets, if applicable. | 5-6 |
|  | 4b | D;V | Specify the key study dates, including start of accrual; end of accrual; and, if applicable, end of follow-up. | 6 |
| Participants | 5a | D;V | Specify key elements of the study setting (e.g., primary care, secondary care, general population) including number and location of centres. | 6 |
|  | 5b | D;V | Describe eligibility criteria for participants. | 6 |
|  | 5c | D;V | Give details of treatments received, if relevant. | NA |
| Outcome | 6a | D;V | Clearly define the outcome that is predicted by the prediction model, including how and when assessed. | 6 |
|  | 6b | D;V | Report any actions to blind assessment of the outcome to be predicted. | NA |
| Predictors | 7a | D;V | Clearly define all predictors used in developing the multivariable prediction model, including how and when they were measured. | 6-7 |
|  | 7b | D;V | Report any actions to blind assessment of predictors for the outcome and other predictors. | NA |
| Sample size | 8 | D;V | Explain how the study size was arrived at. | 9 |
| Missing data | 9 | D;V | Describe how missing data were handled (e.g., complete-case analysis, single imputation, multiple imputation) with details of any imputation method. | 7 |
| Statistical analysis methods | 10a | D | Describe how predictors were handled in the analyses. | 6-7 |
|  | 10b | D | Specify type of model, all model-building procedures (including any predictor selection), and method for internal validation. | 8 |
|  | 10c | V | For validation, describe how the predictions were calculated. | NA |
|  | 10d | D;V | Specify all measures used to assess model performance and, if relevant, to compare multiple models. | 8-9 |
|  | 10e | V | Describe any model updating (e.g., recalibration) arising from the validation, if done. | 9 |
| Risk groups | 11 | D;V | Provide details on how risk groups were created, if done. | 9 |
| Development vs. validation | 12 | V | For validation, identify any differences from the development data in setting, eligibility criteria, outcome, and predictors. | NA |
| **Results** |  |  |  |  |
| Participants | 13a | D;V | Describe the flow of participants through the study, including the number of participants with and without the outcome and, if applicable, a summary of the follow-up time. A diagram may be helpful. | 9 |
|  | 13b | D;V | Describe the characteristics of the participants (basic demographics, clinical features, available predictors), including the number of participants with missing data for predictors and outcome. | 9-10 |
|  | 13c | V | For validation, show a comparison with the development data of the distribution of important variables (demographics, predictors and outcome). | NA |
| Model development | 14a | D | Specify the number of participants and outcome events in each analysis. | 9, Table 1 |
|  | 14b | D | If done, report the unadjusted association between each candidate predictor and outcome. | NA |
| Model specification | 15a | D | Present the full prediction model to allow predictions for individuals (i.e., all regression coefficients, and model intercept or baseline survival at a given time point). | NA |
|  | 15b | D | Explain how to use the prediction model. | 10 |
| Model performance | 16 | D;V | Report performance measures (with CIs) for the prediction model. | 10-11 |
| Model updating | 17 | V | If done, report the results from any model updating (i.e., model specification, model performance). | 11 |
| **Discussion** |  |  |  |  |
| Limitations | 18 | D;V | Discuss any limitations of the study (such as nonrepresentative sample, few events per predictor, missing data). | 15 |
| Interpretation | 19a | V | For validation, discuss the results with reference to performance in the development data, and any other validation data. | NA |
|  | 19b | D;V | Give an overall interpretation of the results, considering objectives, limitations, results from similar studies, and other relevant evidence. | 13-15 |
| Implications | 20 | D,V | Discuss the potential clinical use of the model and implications for future research. | 14-15 |
| **Other information** | | |  |  |
| Supplementary information | 21 | D;V | Provide information about the availability of supplementary resources, such as study protocol, Web calculator, and data sets. | Appendix |
| Funding | 22 | D;V | Give the source of funding and the role of the funders for the present study. | 16 |
